# Nile-Red-Based Fluorescence Probe for Selective Detection of Biothiols, Computational Study, and Application in Cell Imaging

**DOI:** 10.3390/molecules25204718

**Published:** 2020-10-14

**Authors:** Xiang Rong, Zhong-Yong Xu, Jin-Wu Yan, Zhi-Zhong Meng, Bin Zhu, Lei Zhang

**Affiliations:** 1 MOE International Joint Research Laboratory on Synthetic Biology and Medicines, School of Biology and Biological Engineering, South China University of Technology, Guangzhou 510006, China; shirleyrongx@163.com (X.R.); xuzhongyong--728@163.com (Z.-Y.X.); yjw@scut.edu.cn (J.-W.Y.); zzmeng@scut.edu.cn (Z.-Z.M.); 2 Analytical and Testing Center, South China University of Technology, Guangzhou 510640, China

**Keywords:** fluorescent probe, Nile-red, biothiols, living cells, theoretical calculations

## Abstract

A new colorimetric and fluorescence probe **NRSH** based on Nile-red chromophore for the detection of biothiols has been developed, exhibiting high selectivity towards biothiols over other interfering species. **NRSH** shows a blue shift in absorption peak upon reacting with biothiols, from 587 nm to 567 nm, which induces an obvious color change from blue to pink and exhibits a 35-fold fluorescence enhancement at 645 nm in red emission range. **NRSH** displays rapid (<1 min) response for H_2_S, which is faster than other biothiols (>5 min). The detection limits of probe **NRSH** towards biothiols are very low (22.05 nM for H_2_S, 34.04 nM for Cys, 107.28 nM for GSH and 113.65 nM for Hcy). Furthermore, **NRSH** is low cytotoxic and can be successfully applied as a bioimaging tool for real-time monitoring biothiols in HeLa cells. In addition, fluorescence mechanism of probe **NRSH** is further understood by theoretical calculations.

## 1. Introduction

As important reactive sulfur species, small molecular biothiols, such as hydrogen sulfide (H_2_S), cysteine (Cys), homocysteine (Hcy) and glutathione (GSH), play important roles in physiological activities and pathological processes [1,2]. The abnormal levels of intracellular biothiols are closely related to many diseases, including neurotoxicity, cardiovascular diseases, liver damage, Alzheimer’s disease, cancer, and so on [3,4,5]. Therefore, developing hypersensitive methods of detecting biothiols is urgently required, which would be of great assistance to explore the intracellular behavior of biothiols in living organisms and special physiological environments.

Recently, due to high sensitivity and the capability of real-time imaging, fluorescence probes for biothiol detection have attracted more and more attention. A variety of fluorescence probes for biothiols have been devised based on various strategies, including deprotection of 2,4-dinitrobenzenesulfonyl (DNBS), cyclization reaction of aldehyde, Michael addition, thiol-halogen nucleophilic substitution, disulfide exchange reaction and others [6,7,8,9]. Among these strategies, the DNBS-based strategy stands out because of the unique sensitivity and high reactivity of DNBS group towards thiolate anion. Importantly, DNBS can be utilized to design turn-on fluorescence probes since the functional group could totally quench fluorescence [10,11,12,13,14]. On the other hand, multifarious fluorophores have been applied to detect biothiols, including naphtalimide, fluorescein, rhodamine, coumarin, and BODIPY. However, only a few of them could achieve ideal results towards biothiols in the long wavelength region [15,16,17,18,19]. Nile-red (9-(diethylamino)-5*H*-benzo[a]phenoxazin-5-one, **NR**), a dye used to stain in cells, is known to display a blue shift in emission wavelength with increasing hydrophobicity of the environment. Therefore, Nile-red and its derivatives have been used to bioassay because of their high molar-absorption coefficients and long-wavelength emissions [20,21,22,23,24,25,26,27,28]. 

Colorimetric and fluorescent probes have become a new hotspot owing to their remarkable advantages in fundamental study of analytical chemistry and photochemistry [29,30,31,32,33,34,35]. To date, a lot of colorimetric and fluorescent probes for biothiols have been developed [36,37,38,39,40]. Latterly, scientists have begun to design new fluorescent probes based on Nile-red fluorophore for detecting biothiols, and several good probes have been reported [41,42,43,44]. Cui’s group developed a fluorescent probe based on Nile-red for H_2_S detection [43]. The probe exhibits a high sensitivity and selectivity for H_2_S when compared with other small biothiols. Using acrylate group as a reaction site, Ge’s group constructs two NR-based probes for Cys detection [44]. In selectivity experiments, it is found that Hcy and GSH displayed slight fluorescence enhancement, and Cys could induce a remarkable enhancement. 

**Scheme 1:**
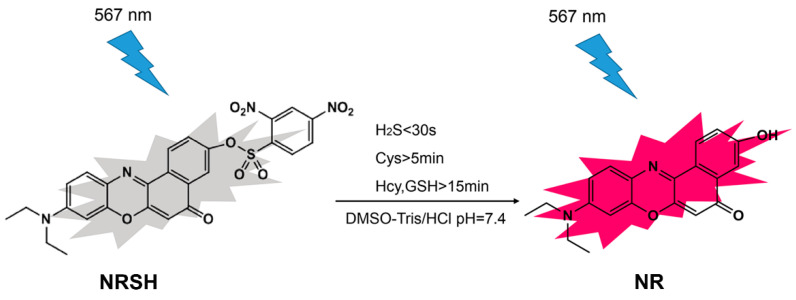
The fluorescence mechanism of NRSH probe.

However, the fluorescent probe based on Nile-red fluorophore for detecting more than one type of biothiols is rarely [45,46]. Herein a new colorimetric and fluorescent probe **NRSH** (2-*O*-(2,4-dinitrobenzenesulfonyl)-9-(diethylamino)-5*H*-benzo[a]phenoxazin-5-one) based on Nile-red fluorophore has been developed to detect the biothiols (Scheme 1). When the probe responds to biothiols, the DNBS group can be removed by mercapto nucleophilic reaction resulting in the generation of **NR** with strong red fluorescence emission. As we expected, **NRSH** can act as a colorimetric and long wavelength fluorescent sensor for biothiols. According to the response times of **NRSH** to biothiols, the probe can be a promising and effective tool to further discriminate H_2_S (<30 s) and Cys (>5 min) from Hcy/GSH (>15 min). In addition, the probe exhibits low detection limits towards biothiols. Most importantly, **NRSH** is low cytotoxic and can be applied as a bioimaging tool for real-time monitoring biothiols in HeLa cells.

## 2. Results and Discussion

### 2.1. Design of the Probe NRSH

**NRSH** has been designed as shown in Scheme 1, and characterized by HRMS and NMR spectroscopy. The detailed spectrum diagrams of structural characterization are shown in the supporting information (Figures S1–S3). The fluorophore **NR** is selected due to a large long wavelength fluorophore and typical intramolecular charge transfer (ICT) process [47,48,49,50], and DNBS is introduced by esterification reaction to form **NRSH**, which might feature a weak fluorescence owing to a characteristic photoinduced electron transfer (PET) process. In the presence of biothiols, such as Cys and GSH etc., DNBS can be removed by mercapto nucleophilic reaction resulting in the generation of **NR** with strong red fluorescence emission at, 645 nm. We anticipate that the probe can be used for detecting biothiols in living cell imaging. In this report, the fluorescent probe **NRSH** shows to be very effective in detecting biothiols, as expected.

### 2.2. Optical Properties

**Figure 1:**
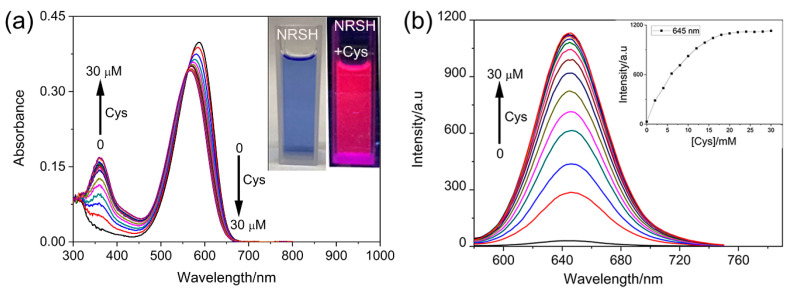
(**a**) Absorption titration of probe (10 μM) toward Cys in DMSO-Tris/HCl buffer (10 mM, pH = 7.4 2:1, *v*/*v*), inset: color changes observed; (**b**) Fluorescence emission of probe toward Cys, inset: plot of the fluorescence intensity of 645 nm with the concentration of Cys; Conditions: incubating in the room temperature for 10 min, slit: excite = emission = 5 nm, λ_ex_ = 567 nm.

The spectral responses of **NRSH** toward Cys were firstly investigated upon the gradual addition of Cys (0–30 μM) in DMSO-Tris/HCl buffer (10 mM, pH = 7.4, 2:1, *v*/*v*). As shown in Figure 1a, **NRSH** shows a maximal absorption peak change from 587 nm (ε = 4.00 × 10^4^ M^−1^ cm^−1^) to 567 nm (ε = 3.42 × 10^4^ M^−1^ cm^−1^) in the above system. Simultaneously, the new absorption peak at 360 nm (ε = 1.69 × 10^4^ M^−1^ cm^−1^) is observed. The solution color changed clearly from blue to pink (Figure 1a inset). Along with the UV-vis changes, **NRSH** displays essential fluorescence emission enhancement at 645 nm upon the addition of Cys. After 2 equiv of Cys was added, the fluorescence is saturated and fluorescence intensity increased about 35-fold (Figure 1b). The responses of **NRSH** toward other biothiols were investigated in the same system as above (Figures S4–S6). The detection limit was calculated using the 3*σ*/*k* method reported in the literature [51], where *σ* is the standard deviation of blank measurement, which was determined from the fluorescence intensity of **NRSH** without biothiols for 11 repetitions, *k* is the slope between the emission intensity of 645 nm and the corresponding concentrations of biothiols). The corresponding detection limits based on the fluorescence titration are 22.05 nM for H_2_S, 34.04 nM for Cys, 107.28 nM for GSH and 113.65 nM for Hcy from 20 blank solutions (Figure S7). Notably, these results indicate that the probe is highly sensitive and may be suitable for detecting biothiols in living systems.

**Figure 2:**
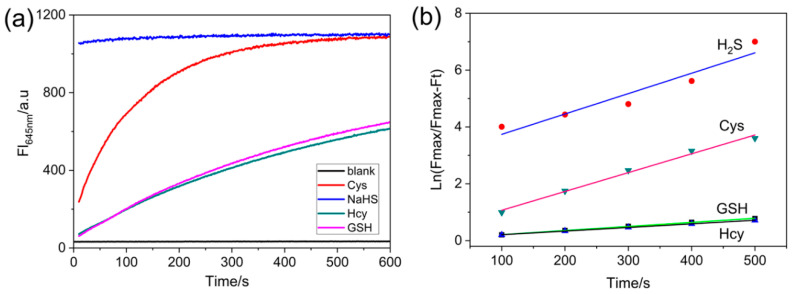
(**a**) Time-dependent fluorescence intensity changes at 645 nm of probe (10 μM) in the absence or presence of biothiols (30 μM) in DMSO-Tris/HCl buffer (10 mM, pH = 7.4 2:1, *v*/*v*); (**b**) The pseudo-first-order kinetic plot of the reaction of probe (10 μM) with biothiols (30 μM). Conditions: incubating in the room temperature for 10 min, slit: excite = emission = 5 nm, λex = 567 nm.

The response times of **NRSH** to biothiols were investigated at room temperature. **NRSH** responds rapidly to H_2_S, and the sensing reaction can complete within 1 min (30 μM NaHS) in DMSO-Tris/HCl buffer. Other biothiols (30 μM), however, cannot complete reaction within 5 min (Figure 2a, Figure S8). The pseudo- first-order rate constants were determined by fitting the fluorescence intensities to the pseudo-first-order equation, and the values are calculated to be 7.17 × 10^−3^ s for H_2_S, 6.63 × 10^−3^ s for Cys, 1.43 × 10^−3^ s for GSH and 1.28 × 10^−3^ s for Hcy (Figure 2b). These results can be attributed to pKa value. The hydrosulfide anion (HS^−^) has low pKa value (~7.0), which is considered to be more nucleophilic than other biothiols in neutral medium; other biothiols have high pKa values (Cys 8.53, GSH 9.20, Hcy 10.00). Therefore, HS^-^ can react faster than other biothiols. 

**Figure 3:**
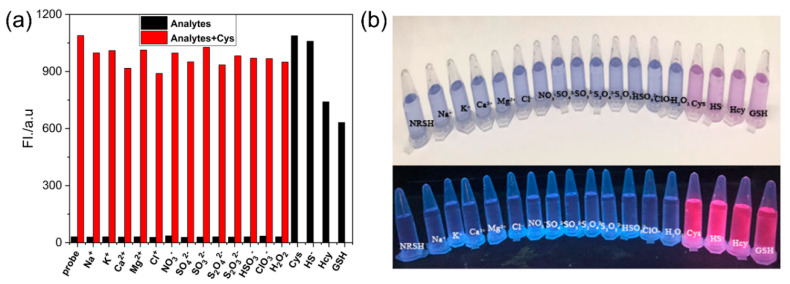
(**a**) Fluorescence responses of **NRSH** (10 μM) in the presence of different analytes (black bars), followed by addition of Cys (30 μM) (red bars); (**b**) Photographs observed after the addition of various analytes to the solution of **NRSH** under nature light (up) and 365 nm UV light (down). Conditions: incubating in the room temperature for 10 min, slit: excite = emission = 5 nm, λ_ex_ = 567 nm.

**Figure 4:**
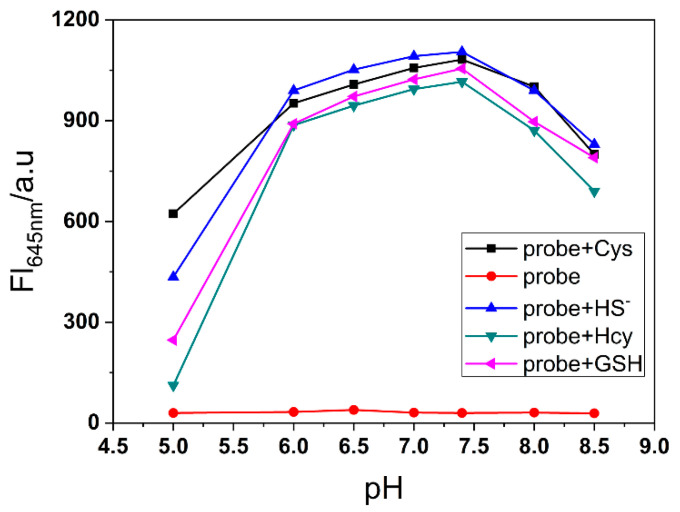
The fluorescence intensity changes of **NRSH** at 645 nm (10 μM) with different pH values in the absence and presence of biothiols (30 μM for H_2_S and Cys; 100 μM for GSH and Hcy). Conditions: incubating in the room temperature for 10 min, slit: excite=emission = 5 nm, λ_ex_ = 567 nm.

To evaluate the selectivity toward biothiols, we measured the fluorescence response of **NRSH** towards other various analytes, and the turn-on fluorescence emission is observed only with the biothiols. Other analytes show negligible fluorescence changes (Figure 3a). These results feature that **NRSH** is highly selective towards biothiols. The digital photograph of probe **NRSH** with different analytes under ambient light and 365 nm UV lamp are shown in Figure 3b. Differentiated colors can be observed by naked eyes under ambient light, and the strong red fluorescence is obtained in addition of biothiols under 365 nm UV lamp. These results indicate that biothiols can be detected through colorimetric and turn-on fluorescence dual channels by probe **NRSH**. Furthermore, the pH effect was measured upon addition of biothiols into **NRSH** in a mixture of DMSO-buffer (2:1 v/v) with pH changing from 5.0 to 8.5 (Figure 4). The fluorescence intensity displays almost no change for **NRSH** alone in a wide pH range. These results indicate that **NRSH** is stable in this pH range. After addition of biothiols, the fluorescence intensity increases dramatically in the pH range of 6.0–8.0. These results indicate that **NRSH** is a promising molecular tool for biological applications under neutral conditions. 

**Scheme 2:**
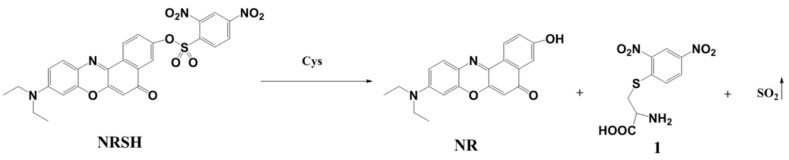
The sensing process of probe **NRSH**.

To confirm the detection mechanism, the absorption and emission spectra of **NR**, **NRSH** and the reaction solution of **NRSH** with Cys (30 μM) were obtained. The maximal absorption peak and the emission peak of reaction solution are in accordance with **NR** (Figure S9). Meanwhile, the reaction products of **NRSH** with Cys were marked in mass spectrum. It gives the mass peaks at *m*/*z* 335.1, which corresponds to [**NR** + H]^+^. On the other hand, the peak at *m*/*z* 288.3 is found, which might be the mass of [**1** + H]^+^ (Scheme 2, Figure S9c). Based on these results, the sensing process of probe **NRSH** to biothiols is most likely the reaction process of thiolysis of the 2,4-dinitrobenzenesulfonyl ester.

### 2.3. Cell Imaging

**Figure 5:**
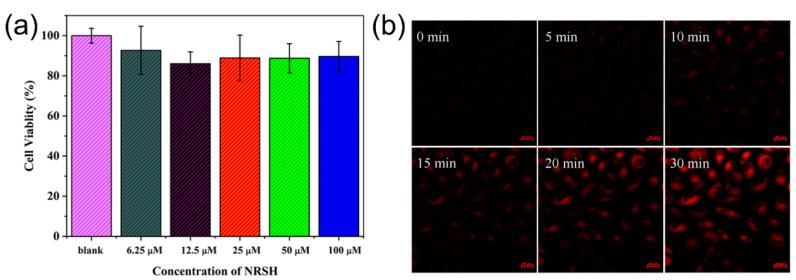
(**a**) Cell viability values (%) estimated by MTT versus different incubation concentrations of **NRSH**. Hela cells were cultured for 48 h in the presence of **NRSH** (6.25, 12.5, 25, 50, 100 μM); The confocal microscopic images of **NRSH** (10 μM) in Hela cells with time: (**b**) The fluorescence imaging of endogenous Cys in living HeLa cells incubated with probe.

Furthermore, we proceeded to validate the feasibility of **NRSH** to image biothiols in living cells. Firstly, the cytotoxicity of **NRSH** was evaluated by an MTT assay in Hela cells incubated with different concentrations of **NRSH** (6.25, 12.5, 25, 50, 100 μM). These results suggest that **NRSH** is low-toxic to Hela cells even for 48 h with the concentration up to 100 μM (Figure 5a). Secondly, the real-time imaging capability of **NRSH** was tested by confocal microscope. It is clearly seen that red fluorescence gradually enhanced in the cells along with the increasing incubation time (Figure 5b). However, no intracellular red fluorescence exists after incubating the same time without probe (Figure S10). Thirdly, we conducted control experiments to trap intracellular thiols with NEM (10 mM). The HeLa cells were pretreated with NEM for 30 min followed by the addition of **NRSH** (10 μM). As expected, cells do show very weak fluorescence signal within the incubation time for 30 min (Figure S11). The living cells were then added Cys (100 μM), and the red fluorescence was quickly observed again within incubation time. These results indicate that the probe **NRSH** can easily penetrate cell membrane resulting in the fluorescent label, and that the fluorescence changes in living cells are indeed caused by biothiols.

### 2.4. Computational Analysis

**Figure 6:**
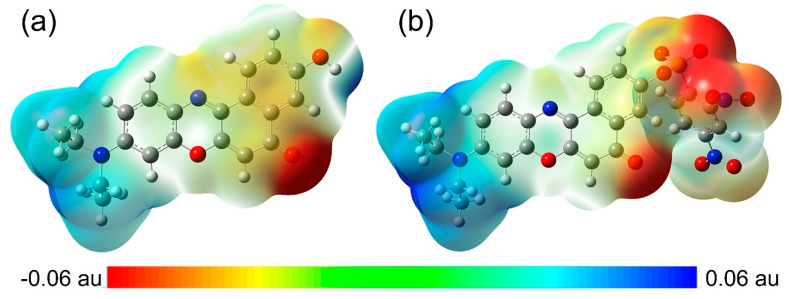
Molecular electrostatic potential (MEP) maps for (**a**) **NR** and (**b**) **NRSH**.

The molecular electrostatic potential (MEP) maps of **NR** and **NRSH** were calculated at DFT/B3YLP/6-311G** level in DMSO medium. MEP is an important parameter to explore intermolecular electrostatic interaction and to predict molecular properties. According to MEP maps in Figure 6, diethylamide moiety always shows blue MEP surface, which represents positive electrostatic potentials. In other words, this group is a strong electron-donating group. On the contrary, DNBS moiety in **NRSH** shows red MEP surface, indicating a strong electron-withdrawing group. Similarly, carbonyl group in two molecules also shows red MEP surface. In summary, all these MEP features are consistent with our design.

**Figure 7:**
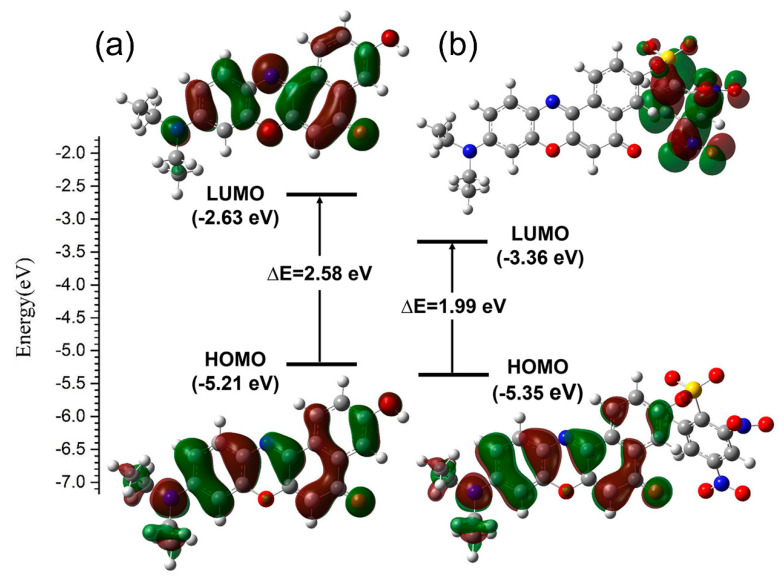
Molecular orbital diagrams and excitation energies of **NR** (**a**) and **NRSH** (**b**).

The electron distribution in the frontier molecular orbitals were accomplished with TD-DFT/B3YLP/6-311G** calculation. And the distribution in HOMO and LUMO are shown in Figure 7. HOMO has the properties of electron donor because of the attraction to its electrons is relatively loose. While LUMO has the properties of electron acceptor because of its electron affinity is relatively strong. It can be easily seen that the contributions to the HOMOs of two compounds are largely located on the backbone of the four connected six-membered rings and diethylamide moieties. The electron densities in the LUMO of **NR** displays a similar distribution of the HOMO, and both the contributions to the two frontier orbitals are located on the whole **NR**, which corresponds to the activated ICT process. However, the contribution to the LUMO of **NRSH** is only located on DNBS group, which coincides with its strong electron-withdrawing property. Consequently, this distributive character indicates that an effective PET process occurs in the excited states of **NRSH** to suppress the fluorescence emission because it has strong quenching moiety DNBS. As a result, the distributive characters of theses frontier molecular orbitals are in accordance with the above optical properties experiments.

The fluorescence emission spectra of **NR** and **NRSH** were accomplished with TD-DFT/B3YLP/6-311G** calculation, and the crucial results are listed in Table 1. According to the Kasha’s rule, fluorophor emits from the lowest state S1 [52]. As shown in Table 1 and Tables S1 and S2, the fluorescence emission wavelength (λ_fluo_) at the first transition state (S_0_→S_1_) of **NR** is predicted at 622 nm (1.99 eV), which is overestimated by 0.07 eV when in comparison with the experimental data (645 nm, 1.92 eV) measured in DMSO. Moreover, the calculated λ_fluo_ value of **NRSH** (1381 nm) is out of the test range (200–900 nm) of the fluorescence spectrophotometer and the oscillator strengths (f) value (0.0001) is evidently less than 0.01, implying that forbidden transition occurs in the excited states of **NRSH**. Therefore, the theoretical calculation results basically conform with the data measured in the experiment. 

**Figure 8:**
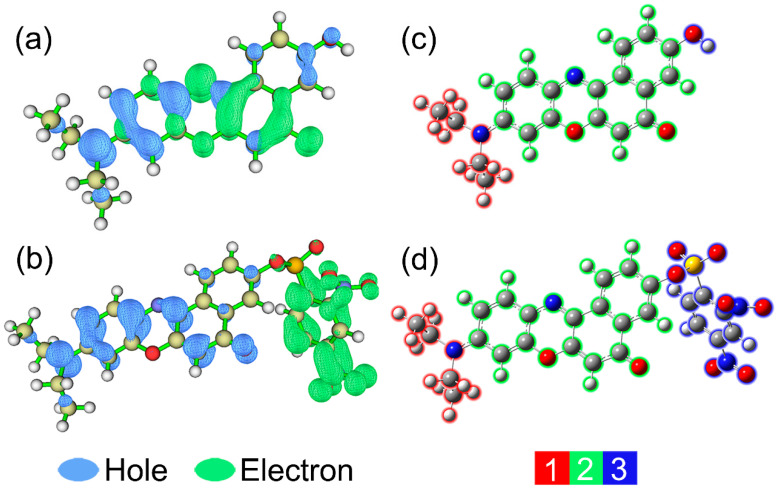
Electron-hole distributions for the S_0_ to S_1_ transitions of (**a**) **NR** and (**b**) **NRSH**; fragment partitions for (**c**) **NR** and (**d**) **NRSH**.

In order to provide a further insight into electron excitation properties, hole-electron distribution analyses were carried out with Multiwfn 3.6 program [53,54]. From the Multiwfn manual, the opinion, “an electron leaves hole and goes to electron”, is used to depict the process of single-electron excitation [53]. As shown in Figure 8a,b (at default isosurface value 0.002), blue and green symbolize the hole distribution and electron distribution, respectively. In addition, the structures of two molecules are divided into 2 or 3 fragments for easy understanding. It can be observed that the trends of hole distributions for **NR** and **NRSH** are very similar from fragment 1 to fragment 2. The figure clearly shows that the overall charge transfer direction is from diethylamide group (fragment 1) to the four connected six membered rings moiety (fragment 2) for **NR**. However, for **NRSH**, almost all electron (99.6%) locates on the fragment 3 (DNBS group). Obviously, the electron excitation characters of **NR** and **NRSH** are corresponding to ICT process and PET process, respectively.

In order to provide a quantitative analysis to figure out how many electrons transfer between different fragments, the interfragment charge transfer (IFCT) analyses were performed with Multiwfn 3.6 program [53], and the results are listed in Table 2 and Table 3. For **NR**, diethylamide (fragment 1) and carbonyl groups (fragment 3) donates 0.22362 and 0.01993 electrons to the four connected six-membered rings moiety (fragment 2), respectively. Therefore, the electron of fragment 2 totally increases by 0.24355 after the electron excitation. While for **NRSH**, fragment 1 and 2 all lose electrons and fragment 4 gains electrons. Notably, fragment 2 decreases by 0.74615 electrons after the electron excitation, which results in the fluorescence quenching. In conclusion, all theoretical calculation results are in good agreement with the experimental results.

## 3. Experimental

### 3.1. Materials and Instrumentations

All solvents and reagents (analytical grade) were obtained commercially and used as received unless otherwise mentioned. Column-layer chromatographic silica gel was purchased from Qingdao Haiyang Chemical Co., Ltd. ^1^H and ^13^C-NMR spectra were collected in DMSO-*d*_6_ at 600 MHz frequency on AVANCE III HD 600 (Bruker Corporation, Rheinstetten, Germany) using TMS as an internal standard. High-resolution mass spectral analyses were carried out using Agilent1290/maXis impact (Bruker Corporation, Hamburg, Germany) at analytical and testing center of South China University of Technology. UV absorption spectra were recorded on a UV-2450 UV-Vis spectrophotometer (Shimadzu Corporation, Kyoto, Japan). Fluorescence measurements were performed using a F-4500 fluorescence spectrophotometer (Hitachi Corporation, Osaka, Japan) equipped with quartz cell of 10 mm path length. **NR** was synthesized by 5-(diethylamino)-2-nitrosophenol hydrochloride and 1,7-dihydroxynaphthalene based on a previously reported method [41].

### 3.2. Synthesis of NRSH

To an anhydrous dichloromethane solution (20 mL) containing **NR** (100 mg, 0.29 mmol) and triethylamine (0.50 mmol, 70 μL) was added dropwise 2,4-dinitrobenzenesulfonyl chloride (89 mg, 0.33 mmol) in dichloromethane (10 mL) at ice bath under the nitrogen atmosphere. The mixture was stirring for 6 h at room temperature, and then water was used to wash the resulting solution triply (30 mL × 3). The dichloromethane phase was dried over Na_2_SO_4_ for one night. After filtration and removal of the organic solvent, the product was purified with silica gel column chromatography by eluting with dichloromethane to yield an atrovirens solid (136 mg, 80%). 

### 3.3. Measurement Procedures

The stock solution of **NRSH** (10 mM) was prepared by dissolving the required amount in analytical grade DMSO. Other analytes including NaCl, Na_2_SO_4_, Na_2_SO_3_, NaHSO_3_, Na_2_S_2_O_3_, Na_2_S_2_O_4_, Cys, Hcy, GSH, NaHS, KCl, MgCl_2_, CaCl_2_, H_2_O_2_ and NaClO were dissolved in deionized water to afford 10 mM aqueous solution. The stock solutions were made freshly and diluted to desirable concentrations. For a typical optical measurement, **NRSH** was diluted to 10 μM in DMSO-Tris/HCl buffer (10 mM, pH = 7.4, 2:1 *v*/*v*). The absorption and fluorescence spectra were recorded at room temperature. The fluorescence spectra were recorded under excitation at 567 nm. The slit size for excitation and emission was 5 nm, respectively. 

### 3.4. Kinetic Studies

The reaction of probe with biothiols in DMSO-Tris/HCl buffer (10 mM, pH = 7.4 2:1, *v*/*v*) was monitored using the fluorescence intensity at 645 nm. The pseudo-first-order rate constant was determined by fitting the fluorescence intensity to the pseudo-first-order equation:

(1) $$\mathrm{Ln}\frac{F_{max}-F_{t}}{F_{max}}=-kt$$

where *F_**max**_* and *F_**t**_* were the fluorescence intensity at 645 nm at the maximum and at *t* time intensity, respectively. *k* was the pseudo-first-order rate constant. The pseudo-first-order plot for the reaction of probe (10 μM) with biothiols (30 μM) was calculated, and the slope of the line provided the pseudo-first-order rate constant.

### 3.5. Cell Image Experiment

HeLa cells were cultured in dulbecco’s modified eagle’s medium supplemented with 10% FBS 100 mg/mL penicillin and 100 mg/mL streptomycin in a 5% CO_2_, water saturated incubator at 37 °C, and then cells were seeded on cover glass bottom confocal dish and continuously incubated at 37 °C in a 5% CO_2_ atmosphere for 24 h (0.5% DMSO was controlled in the cell culture process). The cells were washed three times with PBS buffer solution, and observed by the confocal laser scanning microscopy. Red fluorescence was excited at 565 nm, and the emission was recorded from the red channel (600–750 nm).

### 3.6. Computation Details

The geometry optimization for the ground state structures of **NR** and **NRSH** were accomplished with density functional theory (DFT) calculations at B3YLP/6-311G** level. The frequency analyses were calculated, and no imaginary frequencies were observed, which confirmed that the optimized geometries were the local minima on the potential energy surface. Also, then the time-dependent density functional theory (TD-DFT) calculations were executed for the two optimized structures (Figures S12 and S13) at exited states, and the Cartesian coordinates are available in Tables S3 and S4. Ten lowest singlet states were used to calculate to ensure the accuracy of the results. Multiwfn 3.6 program [53] were used to analyze the charge transfer characteristics. To consider the solvent effect of DMSO, all calculations were in Solvent Model Density (SMD) model [55]. All the calculations mentioned above were implemented by Gaussian 09W program [56].

## 4. Conclusions

In summary, a new colorimetric and fluorescent probe **NRSH** based on Nile-red for the detection of biothiols has been reported. **NRSH** exhibits high sensitivity and selectivity toward biothiols. **NRSH** has faster response time to H_2_S (<1 min) and lower detection limits to biothiols than most reported articles (Table S5). However, the complete reaction of **NRSH** to other biothiols requires at least 7 min in the same conditions. These results may provide a way to distinguish between biothiols. Furthermore, **NRSH** is low-cytotoxic and can be used for real-time detect biothiols by cell fluorescence imaging in living cells, which demonstrates its potential usefulness as a molecular tool for tracking biothiols. We expect that the probe can help us to monitor the level of biothiols in a biological environment and reveal the mysteries of biological processes.

## Figures and Tables

**Table 1:** Calculated fluorescence wavelengths (λ_fluo_), oscillator strengths (*f*), molecular orbital transition contributions to the fluorescence emission spectra and experimental data for the S_0_ → S_1_ excited states of **NR** and **NRSH**.

Probe	Transition	Experiment (nm, eV)	Calculated (nm, eV)	f	%
NR	HOMO→LUMO	645, 1.92	622, 1.99	1.2064	100
NRSH	HOMO→LUMO	–	1381, 0.90	0.0001	100

**Table 2:** Calculated variation of electron population number of each fragment of **NR** and **NRSH**.

	NR	NRSH
1	−0.22455	−0.24376
2	0.24355	−0.74615
3	−0.01900	0.98991

**Table 3:** Calculated net transferred electrons between fragments of **NR** and **NRSH**.

	NR	NRSH
1→2	0.22362	0.00092
1→3	0.00093	0.24284
2→3	−0.01993	0.74707

## Supplementary Materials

The following materials are available online. Figure S1. 1H-NMR spectrum of NRSH. Figure S2. 13C-NMR spectrum of NRSH. Figure S3. HR-MS spectrum of NRSH. Figure S4. a) Fluorescent emission of probe toward GSH; b) plot of the fluorescent intensity of 645 nm with the concentration of GSH. Figure S5. (a) Fluorescent emission of probe toward Hcy; (b) plot of the fluorescent intensity of 645 nm with the concentration of Hcy. Figure S6. (a) Fluorescent emission of probe toward HS^−^; (b) plot of the fluorescent intensity of 645 nm with the concentration of HS^−^. Figure S7. Linear relationship of fluorescence intensity at 645 nm as a function of the concentration of biothiols: (a) Cys, (b) GSH, (c) Hcy, (d) HS^−^. Figure S8. The fluorescence intensity changes at 645 nm of NRSH along with time in the presence of a) Hcy and b) GSH. Figure S9. The UV-vis absorption spectra (a) and the fluorescence spectra (b) of NRSH, NR and the mixture of NRSH and Cys. (c) The MS spectra of reaction mixture of NRSH with Cys. Figure S10. HeLa cells were incubated without probe NRSH. Figure S11. The first row: Hela cells were pretreated with NEM for 30 min and further incubated in the presence of NRSH, the second row: Hela cells were further incubated with Cys after pretreatment with NEM and NRSH. Table S1. Excitation energies and oscillator strengths for NR. Table S2. Excitation energies and oscillator strengths for NRSH: Table S3. Atomic coordinates for NR. Table S4. Atomic coordinates for probe NRSH. Table S5 A comparison about our probe with some reported work.

## References

[1] Li Y., Liu W., Zhang P., Zhang H., Wu J., Ge J., Wang P. (2017). A fluorescent probe for the efficient discrimination of Cys, Hcy and GSH based on different cascade reactions. Biosens. Bioelectron..

[2] Zhang H., Xu L., Chen W., Huang J., Huang C., Sheng J., Song X. (2018). Simultaneous discrimination of cysteine, homocysteine, glutathione, and H_2_S in living cells through a multisignal combination strategy. Anal. Chem..

[3] Chao J., Li M., Liu Y., Zhang Y., Huo F., Yin C. (2019). Fluorescence detection and imaging in zebrafish and Arabidopsis thaliana based on Cys/Hcy breaking space effect. Sens. Actuators B.

[4] Kang J., Huo F., Yao Y., Yin C. (2019). A high signal-to-background ratio H_2_S-specific fluorescent probe based on nucleophilic substitution and its bioimaging for generation H_2_S induced by Ca^2+^ in vivo. Dyes Pigm..

[5] Wang J., Wen Y., Huo F., Yin C. (2019). Based ‘successive’ nucleophilic substitution mitochondrial-targeted H_2_S red light emissive fluorescent probe and its imaging in mice. Sens. Actuators B.

[6] Jiao X., Li Y., Niu J., Xie X., Wang X., Tang B. (2017). Small-molecule fluorescent probes for imaging and detection of reactive oxygen, nitrogen, and sulfur species in biological systems. Anal. Chem..

[7] Jung H.S., Chen X., Kim J.S., Yoon J. (2013). Recent progress in luminescent and colorimetric chemosensors for detection of thiols. Chem. Soc. Rev..

[8] Lee S., Li J., Zhou X., Yin J., Yoon J. (2018). Recent progress on the development of glutathione (GSH) selective fluorescent and colorimetric probes. Coord. Chem. Rev..

[9] Niu L., Chen Y., Zheng H., Wu L., Tung C., Yang Q. (2015). Design strategies of fluorescent probes for selective detection among biothiols. Chem. Soc. Rev..

[10] Hong J., Feng W., Feng G. (2018). Highly selective near-infrared fluorescent probe with rapid response, remarkable large Stokes shift and bright fluorescence for H_2_S detection in living cells and animals. Sens. Actuators B.

[11] Li Y., Wang K., Liu B., Lu X., Li M., Ji L., Mao Z. (2018). Mitochondria-targeted two-photon fluorescent probe for the detection of biothiols in living cells. Sens. Actuators B.

[12] Sedgwick A.C., Gardiner J.E., Kim G., Yevglevskis M., Lloyd M.D., Jenkins A.T.A., Bull S.D., Yoon J., James T.D. (2018). Long-wavelength TCF-based fluorescence probes for the detection and intracellular imaging of biological thiols. Chem. Commun..

[13] Wang L., Yang X.F., Zhao M. (2009). A 4-methylumbelliferone-based fluorescent probe for the sensitive detection of captopril. J. Fluoresc..

[14] Zhang S., Wang Q., Liu X., Zhang J., Yang X.F., Li Z., Li H. (2018). Sensitive and selective fluorescent probe for selenol in living cells designed via a pK_a_ shift strategy. Anal. Chem..

[15] Bu L., Chen J., Wei X., Li X., Ågren H., Xie Y. (2017). An AIE and ICT based NIR florescent probe for cysteine and homocysteine. Dyes Pigm..

[16] Jiang X., Zhang J., Shao X., Zhao W. (2012). A selective fluorescent turn-on NIR probe for cysteine. Org. Biomol. Chem..

[17] Liu K., Shang H., Kong X., Lin W. (2017). A novel near-infrared fluorescent probe with a large Stokes shift for biothiol detection and application in in vitro and in vivo fluorescence imaging. J. Mater. Chem. B.

[18] Meng Y.L., Xin Z.H., Jia Y.J., Kang Y.F., Ge L.P., Zhang C.H., Dai M.Y. (2018). A near-infrared fluorescent probe for direct and selective detection of cysteine over homocysteine and glutathione. Spectrochim. Acta Part A.

[19] Zhang X., Liu J., Ma W., Yang M. (2016). Near-infrared fluorescence of π-conjugation extended benzothiazole and its application for biothiol imaging in living cells. J. Mater. Chem. B.

[20] Black S.L., Stanley W.A., Filipp F.V., Bhairo M., Verma A., Wichmann O., Sattler M., Wilmanns M., Schultz C. (2008). Probing lipid- and drug-binding domains with fluorescent dyes. Biorg. Med. Chem..

[21] Fan J., Guo S., Wang S., Kang Y., Yao Q., Wang J., Gao X., Wang H., Du J., Peng X. (2017). Lighting-up breast cancer cells by a near-infrared fluorescent probe based on KIAA1363 enzyme-targeting. Chem. Commun. Camb..

[22] Jiang X., Guan J., Bian H., Xiao Y. (2017). A cinnamoyl substituted Nile Red-based probe to detect hydrazine. Tetrahedron Lett..

[23] Kong X., Dong B., Zhang N., Wang C., Song X., Lin W. (2017). A unique red-emitting two-photon fluorescent probe with tumor-specificity for imaging in living cells and tissues. Talanta.

[24] Li K., Chen F., Zhang S., Shi W., Han D., Cai C., Chen C.X. (2017). A nile red-based near-infrared fluorescent probe for endogenous hydrogen polysulfides in living cells. Anal. Methods.

[25] Park S., Kubota Y., Funabiki K., Shiro M., Matsui M. (2009). Near-infrared solid-state fluorescent naphthooxazine dyes attached with bulky dibutylamino and perfluoroalkenyloxy groups at 6- and 9-positions. Tetrahedron Lett..

[26] Rostron K.A., Lawrence C.L. (2017). Nile Red Staining of Neutral Lipids in Yeast. Methods Mol. Biol..

[27] Takeshita T., Takeda K., Ota S., Yamazaki T., Kawano S. (2015). A Simple Method for Measuring the Starch and Lipid Contents in the Cell of Microalgae. Cytologia.

[28] Wichmann O., Gelb M.H., Schultz C. (2007). Probing phospholipase A2 with fluorescent phospholipid substrates. ChemBioChem.

[29] Jiang X., Yu H., Zhao J., Sun C., Xie Y., Xiao L. (2015). A colorimetric chemosensor based on new water-soluble PODIPY dye for Hg^2+^ detection. Chin. Chem. Lett..

[30] Lee J., Lee Y.J., Ahn Y.J., Choi S., Lee G.J. (2018). A simple and facile paper-based colorimetric assay for detection of free hydrogen sulfide in prostate cancer cells. Sens. Actuators B.

[31] Murugesan K., Jeyasingh V., Lakshminarayanan S., Govindaraj T.S., Paulraj M.S., Narayanan S., Piramuthu L. (2018). Electron-deficient tripodal amide based receptor: An exclusive turn-on fluorescent and colorimetric chemo sensor for cyanide ion. Spectrochim. Acta Part A.

[32] Tsumura S., Enoki T., Ooyama Y. (2018). A colorimetric and fluorescent sensor for water in acetonitrile based on intramolecular charge transfer: D-(π-A)_2_-type pyridine-boron trifluoride complex. Chem. Commun. Camb..

[33] Wang L., Zhuo S., Cao D. (2017). A colorimetric and fluorescent probe based on michael acceptor type diketopyrrolopyrrole for cyanide detection. J. Fluoresc..

[34] Yang Z., Fu K., Yu J., Zhou P., Cheng Z. (2018). A colorimetric and fluorescent indicator for Hg^2+^ detection based on cinnamamide group-containing rhodamine derivative. J. Fluoresc..

[35] Zhang J., Peng A., Lv Y., Zhang Y., Wang X., Zhang G., Tian Z. (2017). A colorimetric fluorescent probe for SO_2_ derivatives-bisulfite and sulfite at nanomolar level. J. Fluoresc..

[36] Feng S., Fang Y., Feng W., Xia Q., Feng G. (2017). A colorimetric and ratiometric fluorescent probe with enhanced near-infrared fluorescence for selective detection of cysteine and its application in living cells. Dyes Pigm..

[37] Qi S., Liu W., Zhang P., Wu J., Zhang H., Ren H., Ge J., Wang P. (2018). A colorimetric and ratiometric fluorescent probe for highly selective detection of glutathione in the mitochondria of living cells. Sens. Actuators B.

[38] Wang Y., Zhu M., Jiang E., Hua R., Na R., Li Q.X. (2017). A simple and rapid turn on ESIPT fluorescent probe for colorimetric and ratiometric detection of biothiols in living cells. Sci. Rep..

[39] Zhang X., Yan Y., Hang Y., Wang J., Hua J., Tian H. (2017). A phenazine-barbituric acid based colorimetric and ratiometric near-infrared fluorescent probe for sensitively differentiating biothiols and its application in TiO_2_ sensor devices. Chem. Commun..

[40] Zhao B., Yang B., Hu X., Liu B. (2018). Two colorimetric and ratiometric fluorescence probes for hydrogen sulfide based on AIE strategy of alpha-cyanostilbenes. Spectrochim. Acta Part A.

[41] Liu X.D., Fan C., Sun R., Xu Y.J., Ge J.F. (2014). Nile-red and Nile-blue-based near-infrared fluorescent probes for in-cellulo imaging of hydrogen sulfide. Anal. Bioanal. Chem..

[42] Lu J., Song Y., Shi W., Li X., Ma H. (2012). A long-wavelength fluorescent probe for imaging reduced glutathione in live cells. Sens. Actuators B.

[43] Tang C., Zheng Q., Zong S., Wang Z., Cui Y. (2014). A long-wavelength-emitting fluorescent turn-on probe for imaging hydrogen sulfide in living cells. Sens. Actuators B.

[44] Yang X.Z., Wei X.R., Sun R., Xu Y.J., Ge J.F. (2020). Benzoxazine-based fluorescent probes with different auxochrome groups for cysteine detection. Spectrochim. Acta Part A.

[45] Niu H., Ni B., Chen K., Yang X., Cao W., Ye Y., Zhao Y. (2019). A long-wavelength-emitting fluorescent probe for simultaneous discrimination of H_2_S/Cys/GSH and its bio-imaging applications. Talanta.

[46] Lan J.S., Zeng R.F., Liu Y., Xiang Y.W., Jiang X.Y., Liu L., Xie S.S., Ding Y., Zhang T. (2019). A near-infrared Nile red fluorescent probe for the discrimination of biothiols by dual-channel response and its bioimaging applications in living cells and animals. Analyst.

[47] Sarkar N., Das K., Nath D.N., Bhattacharyya K. (1994). Twisted charge transfer processes of Nile red in homogeneous solutions and in faujasite zeolite. Langmuir.

[48] Datta A., Mandal D., Pal S.K., Bhattacharyya K. (1997). Intramolecular Charge Transfer Processes in Confined Systems. Nile Red in Reverse Micelles. J. Phys. Chem. B.

[49] Krishna M.M.G. (1999). Excited-State Kinetics of the Hydrophobic Probe Nile Red in Membranes and Micelles. J. Phys. Chem. A.

[50] Hazra P., Chakrabarty D., Chakraborty A., Sarkar N. (2004). Intramolecular charge transfer and solvation dynamics of Nile Red in the nanocavity of cyclodextrins. Chem. Phys. Lett..

[51] Mei Q., Shi Y., Hua Q., Tong B. (2015). Phosphorescent chemosensor for Hg^2+^ based on an iridium(iii) complex coordinated with 4-phenylquinazoline and carbazole dithiocarbamate. RSC Adv..

[52] Klán P., Wirz J. (2009). Photochemistry of Organic Compounds: From Concepts to Practice.

[53] Lu T., Chen F. (2012). Multiwfn: A multifunctional wavefunction analyzer. J. Comput. Chem..

[54] Liu Z., Lu T., Chen Q. (2020). An sp-hybridized all-carboatomic ring, cyclo [18] carbon: Electronic structure, electronic spectrum, and optical nonlinearity. Carbon.

[55] Marenich A.V., Cramer C.J., Truhlar D.G. (2009). Universal Solvation Model Based on Solute Electron Density and on a Continuum Model of the Solvent Defined by the Bulk Dielectric Constant and Atomic Surface Tensions. J. Phys. Chem. B.

[56] Frisch M.J., Trucks G.W., Schlegel H.B., Scuseria G.E., Robb M.A., Cheeseman J.R., Scalmani G., Barone V., Mennucci B., Petersson G.A. (2010). Gaussian 09, Revision, B.01.

