# Supplementary material for: Nile-Red-Based Fluorescence Probe for Selective Detection of Biothiols, Computational Study, and Application in Cell Imaging
Source: Molecules. 2020 Oct 14;25(20):4718. doi: 10.3390/molecules25204718 (PMC7587360; doi:10.3390/molecules25204718)
Supplement: Supplementary file 1 [file molecules-25-04718-s001.pdf]

Supplementary Information

Nile-red-based Fluorescence Probe for Selective Detection of Biothiols, Computational Study and Application in Cell Imaging

Xiang Rong[1], Zhong-Yong Xu[1], Jin-Wu Yan[1], Zhi-Zhong Meng[1], Bin Zhu[2,*] and Lei Zhang[1,*]

[1] MOE International Joint Research Laboratory on Synthetic Biology and Medicines, School of Biology and Biological Engineering, South China University of Technology, Guangzhou 510006, PR China; shirleyrongx@163.com (X.R.); xuzhongyong--728@163.com (Z.-Y.X.); yjw@scut.edu.cn (J.-W.Y.); zzmeng@scut.edu.cn (Z.-Z.M.)

[2] Analytical and Testing Center, South China University of Technology, Guangzhou 510640, PR China.

* Correspondence: lzhangce@scut.edu.cn (L.Z.); esbzhu@scut.edu.cn (B.Z.); Tel.: +86 (20) 3938-0678 (L.Z.)

Characterization of NRSH

HRMS (ESI): Calcd. for $C_{26}H_{21}N_4O_9S^+$ $[M+H]^+$ m/z 565.1029, found 565.1015; ^1H NMR (600 MHz, DMSO-d_6) δ 9.15 (d, J = 2.3 Hz, 1H), 8.61 – 8.57 (m, 2H), 8.29 (d, J = 8.7 Hz, 1H), 7.88 (d, J = 2.7 Hz, 1H), 7.62 (d, J = 9.1 Hz, 1H), 7.59 (dd, J = 8.8, 2.6 Hz, 1H), 6.88 (dd, J = 9.2, 2.7 Hz, 1H), 6.70 (d, J = 2.7 Hz, 1H), 6.33 (s, 1H), 5.76 (s, 1H), 3.52 (q, J = 7.0 Hz, 4H), 1.17 (t, J = 7.1 Hz, 6H); ^{13}C NMR (151 MHz, DMSO-d_6) δ 180.63, 152.76, 152.10, 151.90, 149.76, 148.64, 147.21, 146.70, 137.02, 134.22, 133.14, 131.75, 131.56, 128.02, 126.84, 125.49, 125.20, 121.59, 118.31, 111.38, 104.86, 96.50, 45.06, 12.93.

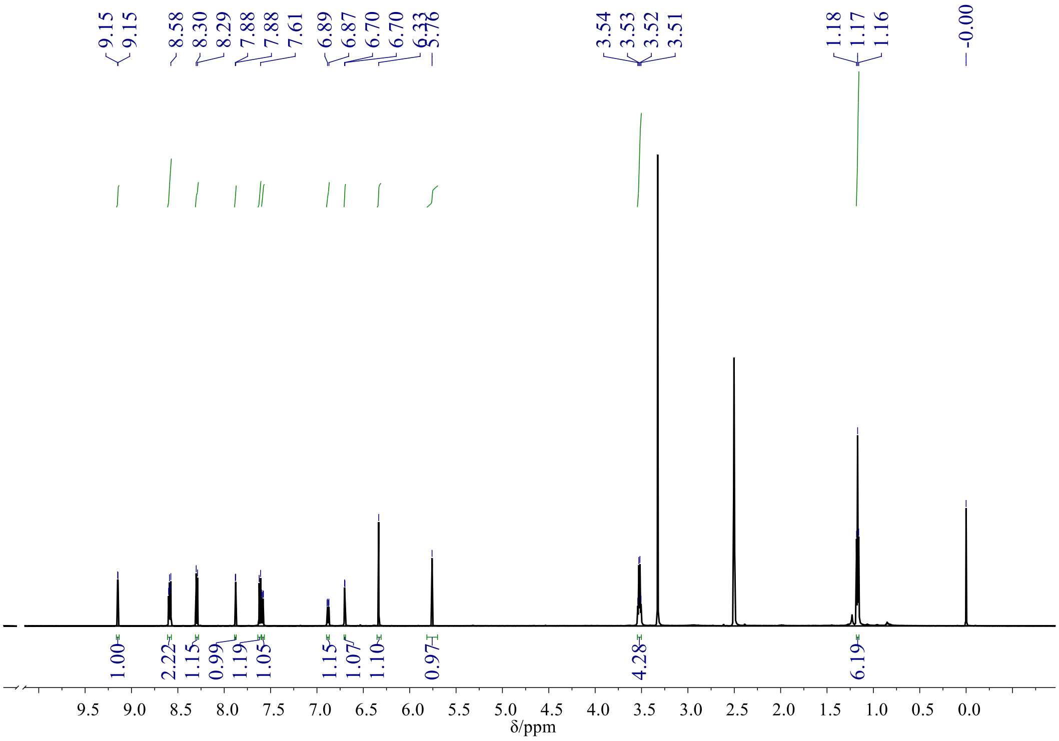

Figure S1. ^1H NMR spectrum of **NRSH** in d_6-DMSO.

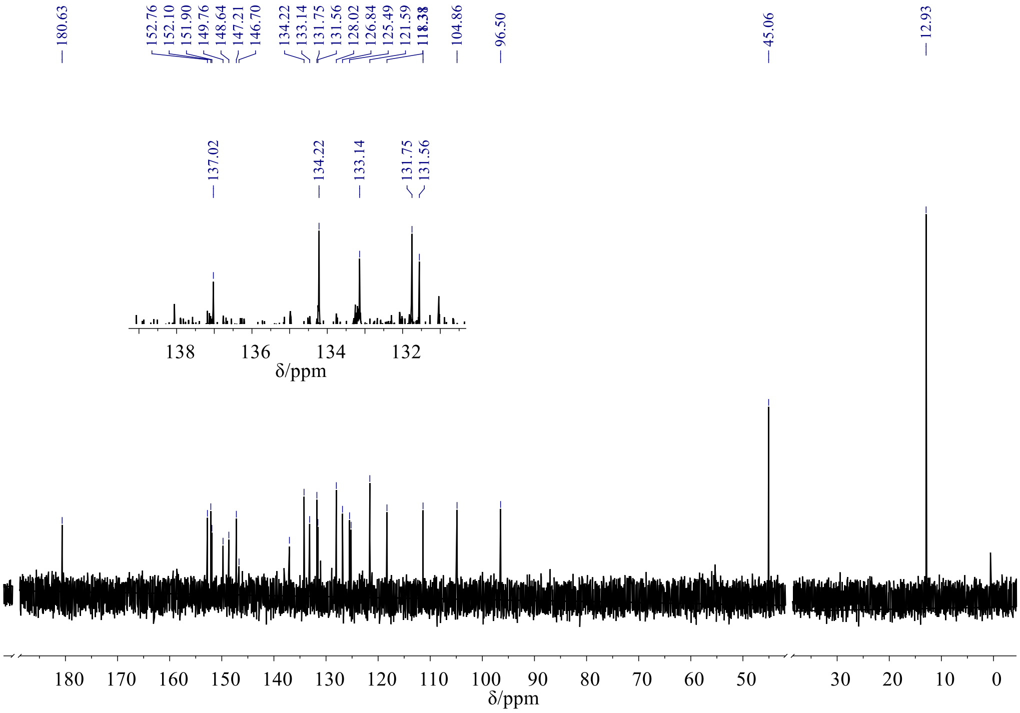

Figure S2. ^{13}C NMR spectrum of **NRSH** in d_6-DMSO.

Acquisition Parameter

Source Type	ESI	Ion Polarity	Positive	Set Nebulizer	0.6 Bar
Focus	Active	Set Capillary	3500 V	Set Dry Heater	180 °C
Scan Begin	50 m/z	Set End Plate Offset	-500 V	Set Dry Gas	4.0 l/min
Scan End	1500 m/z	Set Charging Voltage	0 V	Set Divert Valve	Waste
		Set Corona	0 nA	Set APCI Heater	0 °C

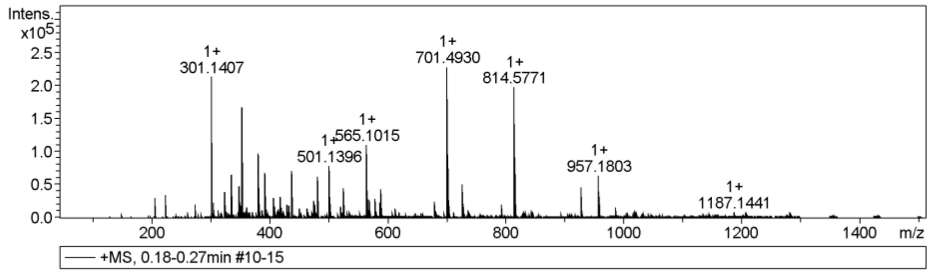

Figure S3. HR-MS spectrum of **NRSH** in methanol.

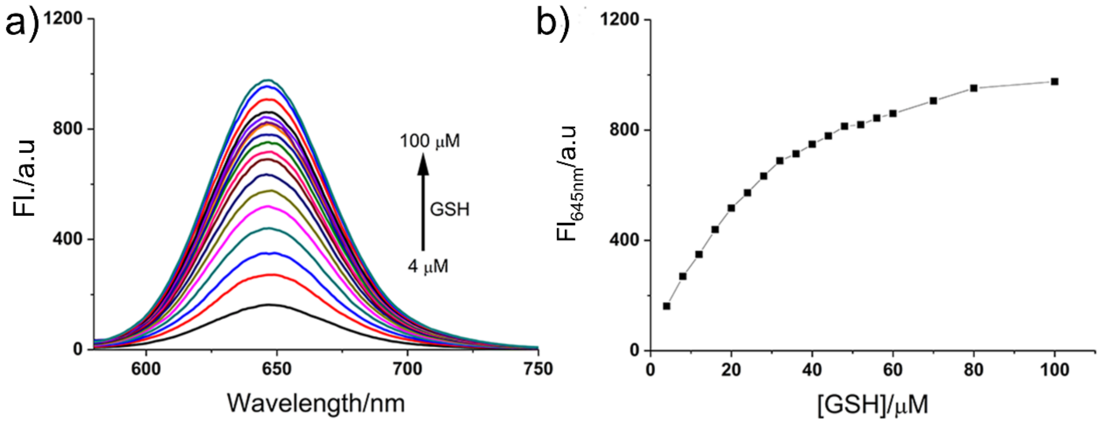

Figure S4. a) Fluorescent emission of probe toward GSH; b) plot of the fluorescent intensity of 645 nm with the concentration of GSH.

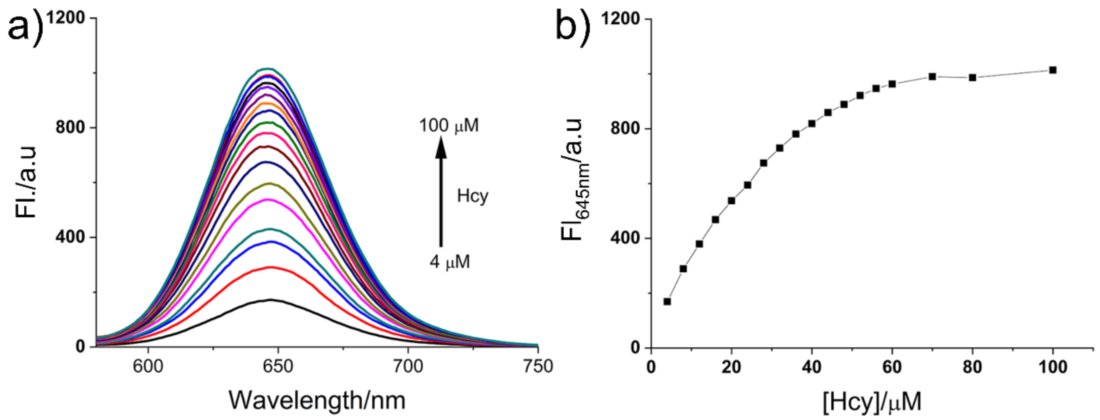

Figure S5. a) Fluorescent emission of probe toward Hcy; b) plot of the fluorescent intensity of 645 nm with the concentration of Hcy.

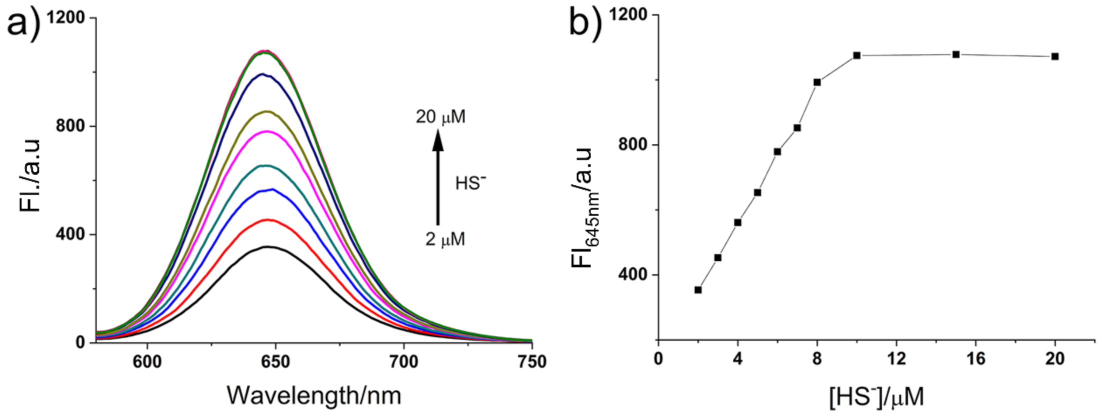

Figure S6. a) Fluorescent emission of probe toward HS^-; b) plot of the fluorescent intensity of 645 nm with the concentration of HS^-.

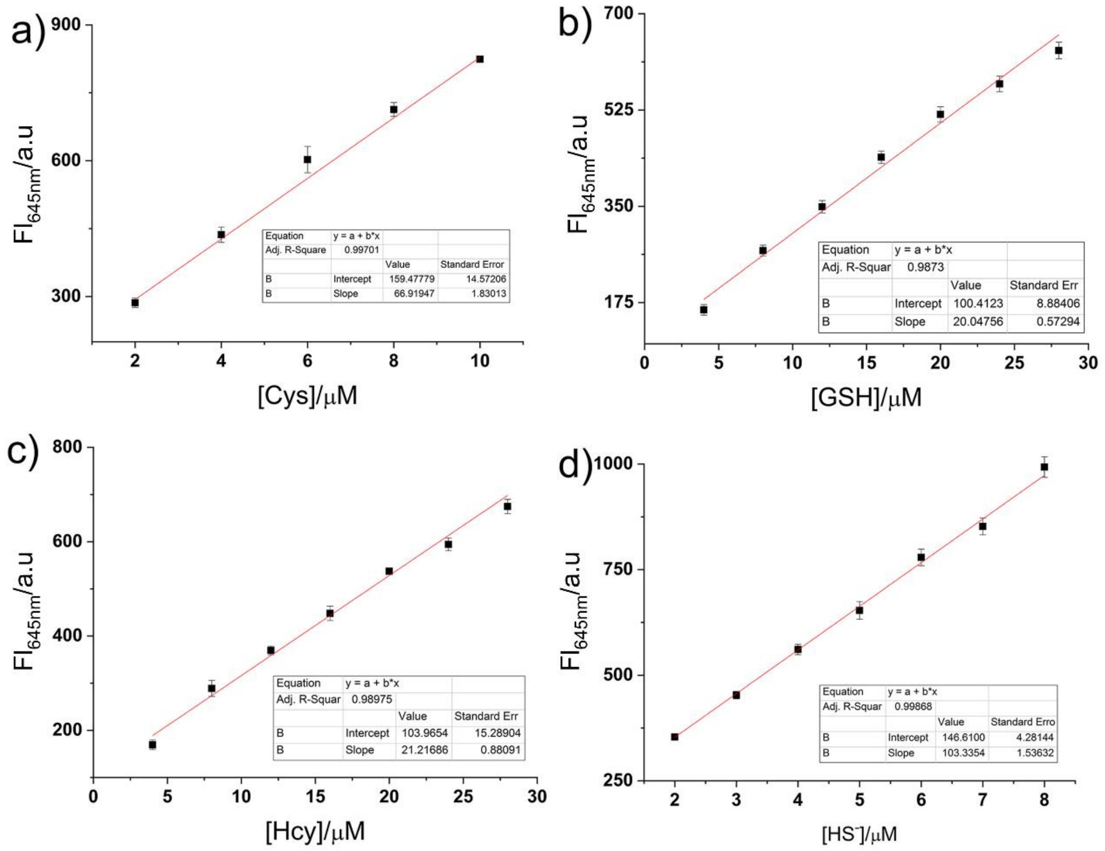

Figure S7. Linear relationship of fluorescence intensity at 645 nm as a function of the concentration of biothiols in DMSO-Tris/HCl buffer (10 mM, pH=7.4 2:1, v/v): a) Cys (2-10 μM), b) GSH, c) Hcy (4-28 μM), d) HS^- (2-8 μM).

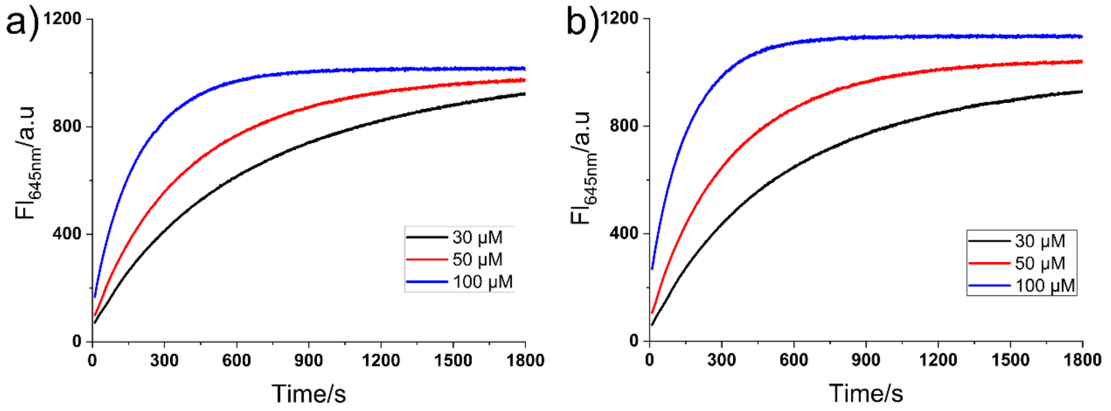

Figure S8. The fluorescence intensity changes at 645 nm of **NRSH** (10 μM) along with time in the presence of a) Hcy and b) GSH.

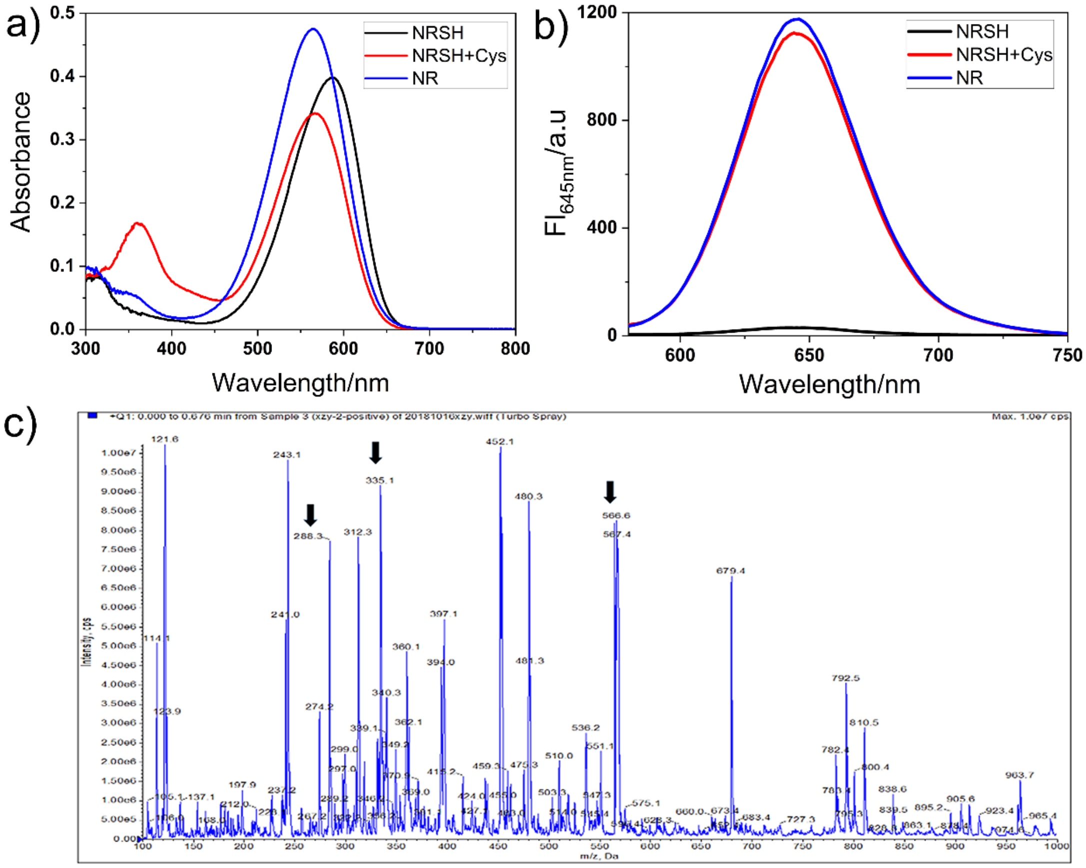

Figure S9. The UV-vis absorption spectra (a) and the fluorescence spectra (b) of **NRSH** (10 μM), **NR** (10 μM) and the mixture of **NRSH** and Cys (30 μM) in DMSO-Tris/HCl buffer (10 mM, pH=7.4 2:1, v/v). c) The MS spectra of reaction mixture of **NRSH** (10 μM) with Cys (30 μM) in methanol.

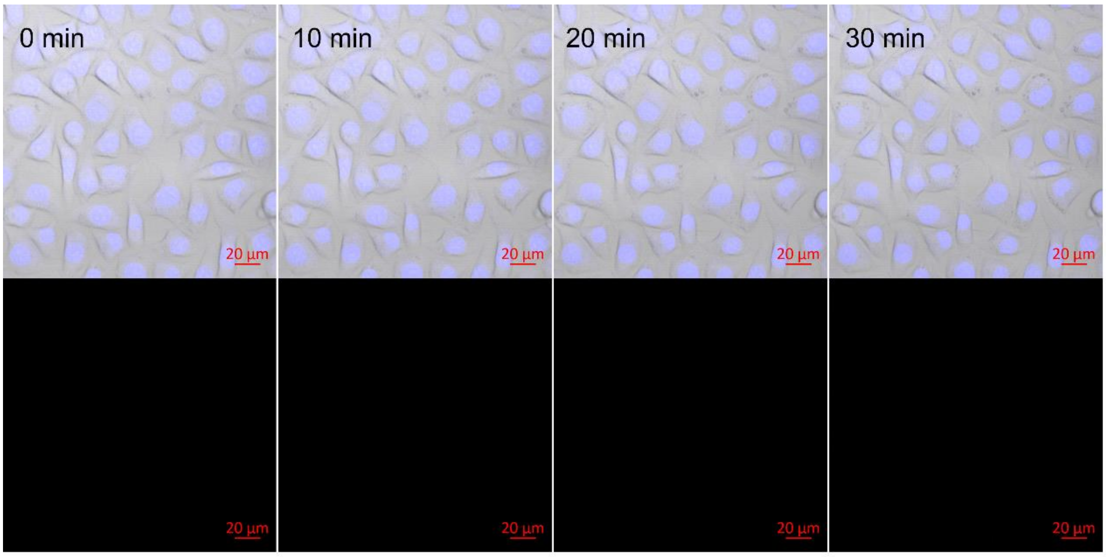

Figure S10. Hela cells were incubated without probe **NRSH**.

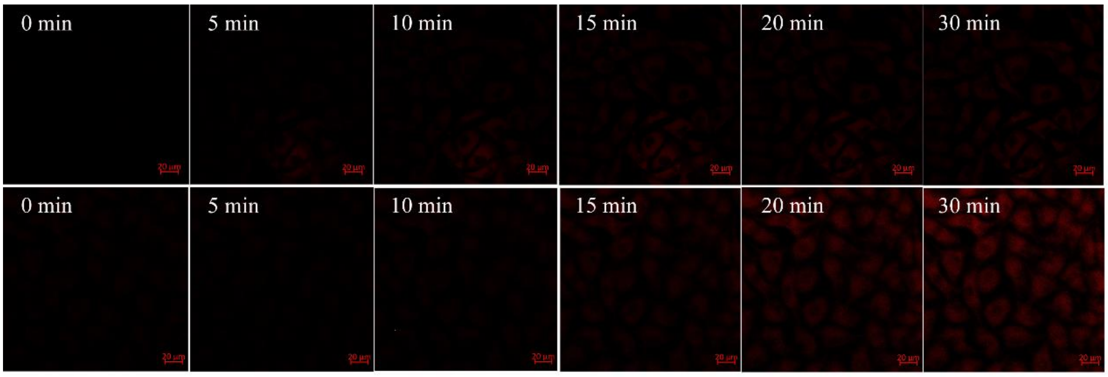

Figure S11. The first row: Hela cells were pretreated with NEM (10 mM) for 30 min and further incubated in the presence of **NRSH** (10 μM), the second row: Hela cells were further incubated with Cys (100 μM) after pretreatment with NEM (10 mM) and **NRSH** (10 μM).

Table S1. Excitation energies and oscillator strengths for **NR**.

Excited State 1: Singlet-A 1.9920 eV 622.42 nm f=1.2064 <S**2>=0.000
88 -> 89 0.70703
This state for optimization and/or second-order correction.
Total Energy, E(TD-HF/TD-DFT) = -1108.37253734
Copying the excited state density for this state as the 1-particle RhoCI density.

Excited State 2: Singlet-A 2.8814 eV 430.30 nm f=0.0155 <S**2>=0.000
87 -> 89 0.69651

Excited State 3: Singlet-A 2.9540 eV 419.72 nm f=0.0000 <S**2>=0.000
85 -> 89 0.69503
85 -> 90 -0.10886

Excited State 4: Singlet-A 3.2707 eV 379.07 nm f=0.0060 <S**2>=0.000
83 -> 89 0.12562
86 -> 89 0.67679

Excited State 5: Singlet-A 3.5785 eV 346.47 nm f=0.0451 <S**2>=0.000
84 -> 89 0.28518
88 -> 90 0.63104

Excited State 6: Singlet-A 3.8046 eV 325.88 nm f=0.0597 <S**2>=0.000
83 -> 89 -0.31113
84 -> 89 0.57314
88 -> 90 -0.21226
88 -> 91 0.12342

Excited State 7: Singlet-A 3.8484 eV 322.17 nm f=0.0036 <S**2>=0.000
82 -> 89 0.69982

Excited State 8: Singlet-A 4.0177 eV 308.59 nm f=0.0838 <S**2>=0.000
83 -> 89 0.51897
84 -> 89 0.27211
86 -> 89 -0.11983
88 -> 90 -0.19050
88 -> 91 -0.29703

Excited State 9: Singlet-A 4.1119 eV 301.52 nm f=0.1690 <S**2>=0.000
83 -> 89 0.31133

88 -> 91 0.59753
88 -> 92 -0.16297

Excited State 10: Singlet-A 4.2871 eV 289.20 nm f=0.1121 <S**2>=0.000
87 -> 90 -0.17271
88 -> 91 0.13336
88 -> 92 0.65229

Table S2. Excitation energies and oscillator strengths for **NRSH**:

Excited State 1: Singlet-A 0.8977 eV 1381.10 nm f=0.0001 <S**2>=0.000
146 ->147 -0.70600
This state for optimization and/or second-order correction.
Total Energy, E(TD-HF/TD-DFT) = -2297.23341243
Copying the excited state density for this state as the 1-particle RhoCI density.

Excited State 2: Singlet-A 1.6982 eV 730.09 nm f=0.0000 <S**2>=0.000
146 ->148 -0.70593

Excited State 3: Singlet-A 2.1382 eV 579.85 nm f=0.1073 <S**2>=0.000
145 ->147 -0.68138
146 ->149 -0.17696

Excited State 4: Singlet-A 2.1446 eV 578.13 nm f=1.2459 <S**2>=0.000
145 ->147 -0.17700
146 ->149 0.68383

Excited State 5: Singlet-A 2.2957 eV 540.06 nm f=0.0001 <S**2>=0.000
144 ->147 0.70624

Excited State 6: Singlet-A 2.6647 eV 465.29 nm f=0.0012 <S**2>=0.000
143 ->147 -0.69226

Excited State 7: Singlet-A 2.8476 eV 435.39 nm f=0.0114 <S**2>=0.000
142 ->147 -0.70250

Excited State 8: Singlet-A 2.9384 eV 421.94 nm f=0.0001 <S**2>=0.000
145 ->148 -0.70339

Excited State 9: Singlet-A 3.0640 eV 404.65 nm f=0.0000 <S**2>=0.000
144 ->148 -0.70611

Excited State 10: Singlet-A 3.1306 eV 396.04 nm f=0.0001 <S**2>=0.000
137 ->147 0.54177
137 ->148 -0.20379
138 ->147 0.35907
138 ->148 -0.13342

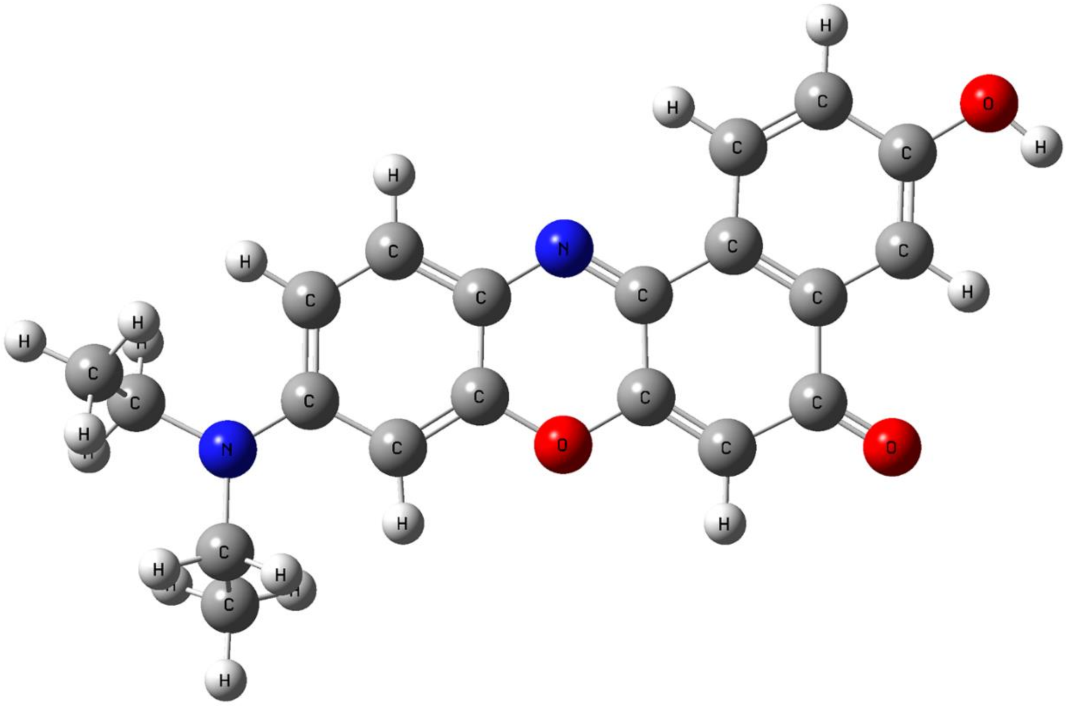

Figure S12. Drawing of **NR** with atoms represented as spheres of arbitrary size (H-white, C-grey, N-blue and O-red) using the GaussView program.

Table S3. Atomic coordinates for **NR**.

Row	Symbol	X	Y	Z
1	C	3.04012	-1.64421	-0.20742
2	C	3.51066	-0.30089	-0.04794
3	C	2.53497	0.72272	0.07397
4	C	1.19581	0.40113	0.03986
5	C	0.72274	-0.91997	-0.11333
6	C	1.69714	-1.93199	-0.23758
7	O	0.29007	1.42245	0.1503
8	C	-1.0464	1.15578	0.115
9	C	-1.47178	-0.22694	-0.02365
10	N	-0.61012	-1.20744	-0.13618
11	C	-1.91315	2.19923	0.21099
12	C	-3.34532	1.99612	0.18762
13	C	-3.82061	0.5849	0.0643
14	C	-2.90838	-0.48567	-0.0379
15	C	-5.19679	0.33943	0.05083
16	C	-5.67695	-0.96	-0.06189
17	C	-4.775	-2.0327	-0.16202
18	C	-3.4148	-1.7938	-0.14996
19	O	-4.14396	2.93502	0.26778
20	O	-7.00299	-1.25768	-0.08025
21	N	4.84252	-0.01734	-0.01191
22	C	5.34346	1.33196	0.2781
23	C	5.50301	2.20739	-0.96633
24	C	5.86871	-1.03976	-0.25467
25	H	3.74539	-2.45796	-0.28865
26	H	2.80685	1.76319	0.17425

27	H	1.35699	-2.95536	-0.34955
28	H	-1.54186	3.21228	0.30783
29	H	-5.87626	1.18148	0.13041
30	H	-5.16206	-3.04142	-0.24755
31	H	-2.72058	-2.62107	-0.22611
32	H	5.90288	3.18562	-0.68325
33	H	6.1965	1.75208	-1.67877
34	H	4.54701	2.36429	-1.47212
35	H	5.50628	-1.74611	-1.00264
36	H	-7.51505	-0.44015	-0.00511
37	H	4.68606	1.81179	1.00501
38	H	6.31057	1.21283	0.77091
39	C	6.31314	-1.77421	1.01159
40	H	6.72347	-0.5303	-0.70462
41	H	6.71624	-1.07499	1.74944
42	H	7.09863	-2.49496	0.76545
43	H	5.48366	-2.31721	1.47157
44	H	5.48366	-2.31721	1.47157

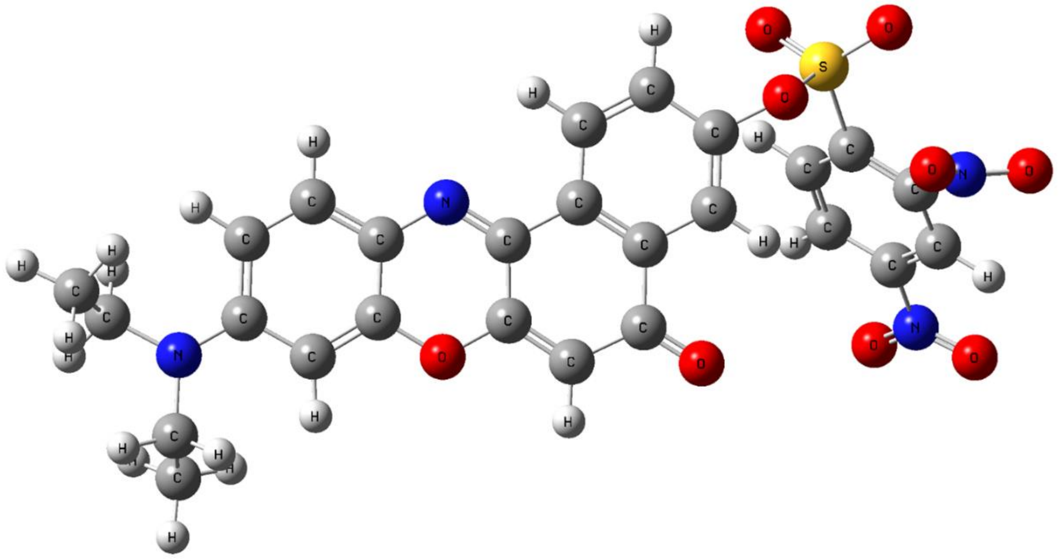

Figure S13. Drawing of **NRSH** with atoms represented as spheres of arbitrary size (H-white, C-grey, N-blue, S-yellow and O-red) using the GaussView program.

Table S4. Atomic coordinates for probe **NRSH**.

Row	Symbol	X	Y	Z
1	C	6.589887	-1.119865	-0.952768
2	C	6.943041	0.091241	-0.27028
3	C	5.904111	0.794072	0.397195
4	C	4.619922	0.301998	0.372991
5	C	4.262684	-0.892132	-0.295433
6	C	5.299193	-1.584499	-0.958984
7	O	3.649043	1.015002	1.022868
8	C	2.360319	0.56917	1.024952

9	C	2.06057	-0.667009	0.329418
10	N	2.984418	-1.353363	-0.299368
11	C	1.426088	1.310752	1.680177
12	C	0.041114	0.902428	1.729816
13	C	-0.304905	-0.367714	1.023113
14	C	0.673936	-1.127543	0.348194
15	C	-1.63422	-0.803424	1.040388
16	C	-1.96999	-1.974346	0.391235
17	C	-1.018112	-2.749922	-0.274478
18	C	0.296801	-2.320659	-0.294181
19	O	-0.823948	1.556834	2.320215
20	O	-3.298177	-2.439105	0.487584
21	N	8.223049	0.546902	-0.260239
22	C	8.626302	1.72549	0.519753
23	C	8.469108	3.042244	-0.242192
24	C	9.295971	-0.104206	-1.026195
25	H	7.35008	-1.695318	-1.459536
26	H	6.084551	1.7235	0.916466
27	H	5.047859	-2.5055	-1.472705
28	H	1.709905	2.227522	2.182668
29	H	-2.378044	-0.221115	1.568067
30	H	-1.310437	-3.671868	-0.760343
31	H	1.05022	-2.906759	-0.80384
32	H	8.825526	3.872307	0.374775
33	H	9.053829	3.03246	-1.1662
34	H	7.424804	3.234983	-0.499937
35	H	8.89682	-0.470609	-1.972755
36	H	8.064523	1.750196	1.454731
37	H	9.67287	1.580032	0.793843
38	C	9.995449	-1.228334	-0.260352
39	H	10.017775	0.674572	-1.279046
40	H	10.426074	-0.857678	0.673906
41	H	10.807528	-1.639045	-0.867468
42	H	9.30472	-2.040793	-0.021641
43	S	-4.353455	-2.171062	-0.741894
44	C	-4.687603	-0.391342	-0.682294
45	C	-4.085263	0.385765	-1.666545
46	C	-5.606606	0.202008	0.193366
47	C	-4.37258	1.744186	-1.76561
48	H	-3.400926	-0.068568	-2.369231
49	C	-5.934497	1.541431	0.08596
50	C	-5.294372	2.2929	-0.890278
51	H	-3.896153	2.353084	-2.520607
52	H	-6.65528	1.990224	0.75429
53	O	-3.686798	-2.437067	-2.004653
54	O	-5.548799	-2.897225	-0.365178
55	N	-6.252654	-0.536523	1.300921
56	O	-5.529817	-1.203065	2.022659
57	O	-7.457175	-0.390131	1.435817
58	N	-5.621723	3.734577	-0.995611

59	O	-6.480564	4.17659	-0.245575
60	O	-5.012599	4.392926	-1.825844

Table S5 A comparison about our probe with some reported work

probe	Analytes	Time	LOD (μM)	λ_{em}(nm)	Reference
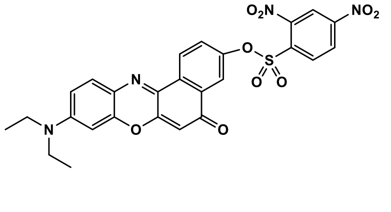	H_2S	< 1 min	0.022	645	This work
	Cys	> 5 min	0.034		
	Hcy	> 15 min	0.107		
	GSH	> 15 min	0.114		
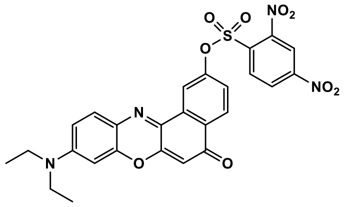	GSH	10 min	0.043	650	[1]
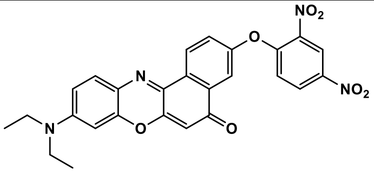	H_2S	10 min	0.023	656	[2]
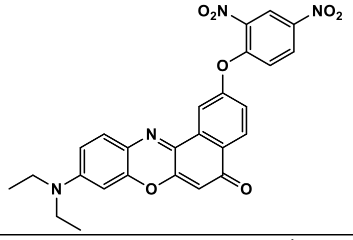	H_2S	20 min	0.27	655	[3]
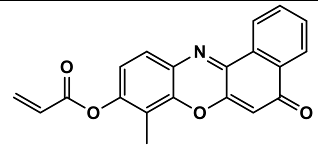	Cys	15 min	0.0198	631	[4]
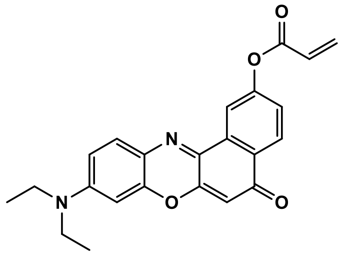	Cys	5 min	0.145	665	[4]
	H_2S	2 min	0.04	650	[5]

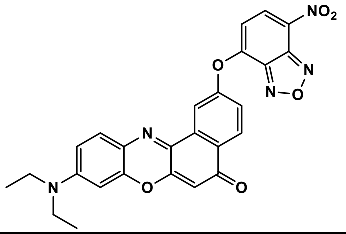	Cys	< 30 min	0.09	550, 650	
	Hcy	< 30 min	0.30	550, 650	
	GSH	> 60 min	0.24	650	

References

1. Lu, J.; Song, Y.; Shi, W.; Li, X.; Ma, H. A long-wavelength fluorescent probe for imaging reduced glutathione in live cells. *Sens. Actuators, B* **2012,** *161 (1)*, 615-620.
2. Liu, X.D.; Fan, C.; Sun, R.; Xu, Y.J.; Ge, J.F. Nile-red and Nile-blue-based near-infrared fluorescent probes for in-cellulo imaging of hydrogen sulfide. *Anal. Bioanal. Chem.* **2014,** *406* (28), 7059-7070.
3. Tang, C.; Zheng, Q.; Zong, S.; Wang, Z.; Cui, Y. A long-wavelength-emitting fluorescent turn-on probe for imaging hydrogen sulfide in living cells. *Sens. Actuators, B* **2014**, *202*, 99-104.
4. Yang, X.Z.; Wei, X.R.; Sun, R.; Xu, Y.J.; Ge, J.F. Benzoxazine-based fluorescent probes with different auxochrome groups for cysteine detection. *Spectrochim. Acta, Part A* **2020,** *226*, 117582.
5. Lan, J.S.; Zeng, R.F.; Liu, Y.; Xiang, Y.-W.; Jiang, X.Y.; Liu, L.; Xie, S.S.; Ding, Y.; Zhang, T. A near-infrared Nile red fluorescent probe for the discrimination of biothiols by dual-channel response and its bioimaging applications in living cells and animals. *Analyst* **2019,** *144* (11), 3676-3684.
